# Enhancing drought, heat shock, and combined stress tolerance in Myrobalan 29C rootstocks with foliar application of potassium nitrate

**DOI:** 10.1186/s12870-024-04811-4

**Published:** 2024-02-27

**Authors:** Ibrahim Bolat, Kubra Korkmaz, Meral Dogan, Metin Turan, Cengiz Kaya, Hanifeh Seyed Hajizadeh, Ozkan Kaya

**Affiliations:** 1 Faculty of Agriculture, Department of Horticulture, Harran University, Sanliurfa, Türkiye; 2 Graduate School of Natural and Applied Sciences, Department of Horticulture, Harran University, Sanliurfa, Türkiye; 3 Faculty of Economy and Administrative Science, Yeditepe University, Istanbul, 34755 Türkiye; 4 Soil Science and Plant Nutrition Department, Harran University, Sanliurfa, Türkiye; 5 Department of Horticulture, Faculty of Agriculture, University of Maragheh, Maragheh, 55136-553 Iran; 6 Republic of Turkey Ministry of Agriculture and Forestry, Erzincan Horticultural Research Institute, Erzincan, 24060 Türkiye; 7 Department of Plant Sciences, North Dakota State University, Fargo, ND 58102 USA

**Keywords:** Heat shock stress, Drought stress, Rootstock, Recovery, Potassium nitrate

## Abstract

**Background:**

Drought and heat stress are significant concerns to food security in arid and semi-arid regions, where global warming is predicted to increase both frequency and severity. To cope with these challenges, the use of drought-tolerant plants or technological interventions are essential. In this study, the effects of foliar potassium nitrate (KNO_3_) application on the stress tolerance and recovery of Myrobalan 29C rootstocks (*Prunus cerasifera* Ehrh.) were evaluated. These rootstocks are widely recognized for their adaptability and are extensively used in fruit production. To assess their response, the rootstocks were subjected to drought, heat shock, or a combination of both stressors. Additionally, they were treated with 1.0% KNO_3_ via foliar application. Throughout the stress and recovery periods, various morphological, physiological, and bio-chemical parameters were measured.

**Results:**

Based on our results, KNO_3_ treatment improved LRWC, Chl stability, SC, and key stress markers like proline, MDA, H_2_O_2_, along with antioxidant enzymes CAT, SOD, POD during both stress and recovery phases. Moreover, our results emphasized KNO_3_'s critical role in hormone regulation under stress. KNO_3_ application significantly altered hormone levels, notably increasing ABA during drought and heat shock stress, essential for stress response and adaptation. In contrast, IAA, GA, and cytokinin’s significantly increased during the recovery phase in KNO_3_-treated plants, indicating improved growth regulation and stress recovery. In addition, KNO_3_ application improved the recovery process of the rootstocks by restoring their physiological and biochemical functions.

**Conclusion:**

This study suggests that the application of foliar KNO3 is an effective technique for enhancing the drought and heat tolerance as well as the recovery of Myrobalan 29C rootstocks. These results hold significant value for farmers, policymakers, and researchers, as they offer crucial insights into the development of drought-tolerant crops and the management of climate change’s adverse effects on agriculture.

## Introduction

Global warming poses a profound and imminent threat, particularly in arid and semi-arid regions. The increasing frequency and intensity of droughts and heat waves are clear indicators of the challenges we face [1]. Rising temperatures and prolonged periods of drought disrupt crop growth, affecting yield and quality [2]. Heat stress on plants, combined with water scarcity, jeopardizes the survival of crops and livestock [3]. These abiotic stresses can severely impair plant development and yield by interfering with biochemical and physiological events, including photosynthesis, water balance, nutrient uptake, and antioxidant defense [4, 5]. Therefore, developing and breeding drought- and heat-tolerant plants is crucial to reduce the deleterious impacts of climate change on agriculture.

Plants have developed intricate strategies to adapt and withstand the challenges posed by drought and heat stress. These adaptive responses enable them to sustain their physiological functions and thrive even in unfavourable environmental conditions [6]. These adaptive strategies involve complex physiological, biochemical, and molecular responses. Understanding these mechanisms is crucial for developing effective strategies to enhance the drought tolerance of crops. Osmotic regulation plays a crucial role in the plant stress response, serving as a key mechanism for plants to manage water stress. By accumulating compatible osmolites, including proline, plants can maintain cell turgor and prevent water loss. The osmotic adjustment process helps plants sustain cellular hydration and minimize damage caused by water deficit [5, 7]. Another crucial aspect of the plant stress response is the initiation of antioxidant defense systems. Under stress conditions, the accumulation of reactive oxygen species (ROS) can induce oxidative harm to cellular constituents. In response to this challenge, plants employ several strategies. They boost the activity and expression of antioxidant enzymes as a means to counteract the harmful effects of ROS [8]. Simultaneously, non-enzymatic antioxidants and phenolics play crucial roles in scavenging ROS and safeguarding plant cells against oxidative stress [4, 5].

Plant adaptation to drought and heat stress is significantly influenced by hormonal regulation. Signalling triggered by drought and heat shock can lead to alterations in the metabolic routes of plant hormones, specifically abscisic acid (ABA). ABA induction stimulates the modulation of stomatal closure through gene expression, leading to a decrease in water loss [9]. In addition to ABA, cytokinins and indole-3-acetic acid (IAA) also serve as key players in plant growth, development, and stress adaptation. Cytokinins facilitate cell division and delay senescence [10], while IAA regulates cell elongation and differentiation [11]. Maintaining a delicate balance among these hormones is essential for optimal plant growth and effective stress adaptation [12]. The strategies involve hormonal regulation in plants, particularly through hormones such as ABA, Ethylene, Auxins, Cytokinins, Gibberellins, and Salicylic Acid (SA), each playing a role in growth, development, and stress adaptation. However, these strategies may not be sufficient to mitigate the combined impacts of drought and heat stress on plants. Therefore, there is a need to explore alternative techniques or practices that can enhance the stress tolerance and recovery potential of plants, such as the implementation of advanced genetic engineering to develop stress-resilient plant varieties or the adoption of novel agronomic practices aimed at improving soil health and water retention. One such technique is the application of potassium nitrate (KNO_3_) as a foliar treatment, which has been shown to enhance the osmotic regulation and antioxidant capacity of citrus plants under water stress [13]. However, the effects of KNO_3_ on plants under the co-occurring challenges of drought and heat stress are not well understood.

Myrobalan 29C rootstocks (*Prunus cerasifera* Ehrh.) are widely used in fruit production because they can adapt to different soil and climatic conditions [14]. However, they still face the challenge of drought and heat stress, which can impair their growth and performance. These rootstocks, however, remain susceptible to the synergistic stresses of drought and heat, which markedly compromise their growth and overall performance. Addressing the critical challenge of escalating climatic variability requires pioneering innovative agricultural strategies to enhance the resilience of Myrobalan 29C rootstocks (*Prunus cerasifera* Ehrh.), fundamental to fruit production due to their versatility across diverse soil and climate conditions. Indeed, it is imperative to not only highlight the restorative benefits of KNO_3_ treatment but also to address a crucial gap in existing research by offering a comprehensive examination of the physiological responses to the combined stresses of drought and heat. In this study, therefore, we explored the potential of foliar potassium nitrate (KNO_3_) application in enhancing the stress tolerance and recovery of Myrobalan 29C rootstocks under drought, heat, or their combined effects. Our objective was to address the following research questions while hypothesizing that KNO_3_ application would improve the osmotic adjustment, antioxidant defense, and hormonal balance of Myrobalan 29C rootstocks under these challenging conditions:(i) How to do drought and heat stress impact the morphology and physiology of Myrobalan 29C rootstocks? (ii) How does the application of KNO_3_ improve the stress tolerance and recovery of Myrobalan 29C rootstocks? and (iii) What are the potential mechanisms underlying the beneficial effects of KNO_3_ on Myrobalan 29C rootstocks?

To test this hypothesis, we measured various parameters related to plant growth, water status, photosynthesis, antioxidant defence, hormonal balance, and mineral nutrition in Myrobalan 29C rootstocks treated with potassium nitrate (KNO_3_) under conditions of drought, heat stress, or their combined effects. Additionally, the responses of these parameters during stress and recovery periods were compared. The findings of this study yield crucial insights into the improvement of drought and heat tolerance in Myrobalan 29C rootstocks through the application of KNO_3_ treatment.

## Materials and methods

### Plant growth conditions and treatments

The experiment was carried out during the 2021 season in a glass greenhouse situated at the Harran University Faculty of Agriculture (38º 59' E longitude, 37º 10' N latitude, with an elevation of 530 m above sea level), using Myrobalan 29C (*Prunus cerasifera* Ehrh.) rootstock. The Myrobalan 29C rootstock used in our study were purchased from Elma Tarim (Isparta/Turkey). The selection of Myrobalan 29C rootstock was based on its widespread use in fruit production and its proven adaptability to various soil and climatic conditions [14]. The greenhouse provided controlled environmental conditions, maintaining a temperature range of 25 ± 2 °C during the daytime and 21 ± 2 °C during the night. The greenhouse also ensured photosynthetically active radiation (PAR) ranging from 650 to 840 mmol m^−2^ s^−1^ and relative humidity of 50 ± 5%.

For the experiment, one-year-old woody cuttings of Myrobalan 29C rootstocks were transferred to a rooting medium, and after rooting occurred, uniformly growing rootstocks were selected and planted directly into plastic pots. Consequently, the initial methodology for selecting rootstocks characterized by uniform growth for their direct transplantation into plastic pots involved the delineation of a specific growth length range. This was operationalized by choosing rootstocks that demonstrated a growth length between 10–15 cm, a measure instituted to guarantee both consistency and uniformity across the experimental setup. Each plastic pot was filled with a mixture of peat moss (Klasmann, TS1, Klasmann-Deilman Gmbh, Lower Saxony, Germany) and 1.0 kg m^3^ of 14:10:18 N:P: K fertilizer, ensuring an electrical conductivity of 35 mS m^−1^ and a pH of 6.5. One cutting of Myrobalan 29C rootstock was planted in each pot. This planting procedure was carried out on March 15. The pots were sealed with plastic film to shield the soil to minimize surface evaporation. The experiment followed a split-plot experimental design with six replications at random, with one plant assigned to each replicate. The experiment was structured with two main plot treatments: Non-KNO_3_ (no potassium nitrate) and 1.0% KNO_3_ (with potassium nitrate). Within these main plots, the sub-plots were designated based on four different stress conditions: control (no stress), drought, heat shock, and drought + heat shock (Table 1).

**Table 1:** A visual representation of different experimental treatments, illustrating the various combinations of KNO_3_ application and stress conditions (drought, heat shock, and both combined) administered to Myrobalan 29C rootstocks

**Treatments**	**Description**
Non-KNO_3_-Control	No KNO_3_ application, no stress
Non-KNO_3_-Drought	No KNO_3_ application, drought stress
Non-KNO_3_ + Heat Shock	No KNO_3_ application, heat shock stress
Non-KNO_3_-Drought + Heat Shock	No KNO_3_ application, combined drought and heat shock stress
KNO_3_-Control	KNO_3_ application, no stress
KNO_3_-Drought	KNO_3_ application, drought stress
KNO_3_-Heat Shock	KNO_3_ application, heat shock stress
KNO_3_-Drought + Heat Shock	KNO_3_ application, combined drought and heat shock stress

To maintain soil moisture, plants were irrigated equally until the start of the treatments, with the irrigation frequency adjusted to maintain a 100% field capacity. All pots were manually applied with 50% Hoagland solution, a widely used hydroponic nutrient solution for plant growth, every seven days until the start of experimental treatments. The composition of the Hoagland solution used in the study included all macro and micronutrients [15]. Two weeks before the stress treatments, a 1.0% KNO_3_ solution was applied to the leaves of the plants, while the control plants were treated with distilled water alone. During the stress period, plants exposed to control and heat shock conditions were irrigated every two days, while plants experiencing drought alone and combined with heat shock conditions were deprived of irrigation for a duration of 15 days. After the stress period, a heat shock treatment was administered to half of the plants in each group, where they were transferred to a heat chamber and exposed to a temperature of 43 ± 1 °C for 2 h. Within the heat shock chamber, the photosynthetically active radiation (PAR) levels were meticulously regulated to align with those established in the main greenhouse area, ensuring uniform photosynthetic activity across all treatment groups. This uniformity in PAR levels, alongside consistent relative humidity, was essential for attributing variations in physiological and biochemical responses solely to the thermal stress induced by the heat shock treatment. By maintaining these environmental parameters constant, we could isolate the effect of elevated temperature, allowing for a clear analysis of its impact on plant physiology. This strategic control over experimental conditions was fundamental to our investigation, enabling us to discern the specific physiological responses elicited by heat stress, distinctly from other potential environmental influences.

To examine the effects of stress on the rootstocks, we subjected them to two weeks of stress treatments and collected data for analysis from three rootstocks per treatment. After the stress treatment, we allowed the remaining three rootstocks per treatment to recover under normal conditions for 10 days. We then performed a second sampling for further analysis at the end of the recovery stage.

**Fig. 1:**
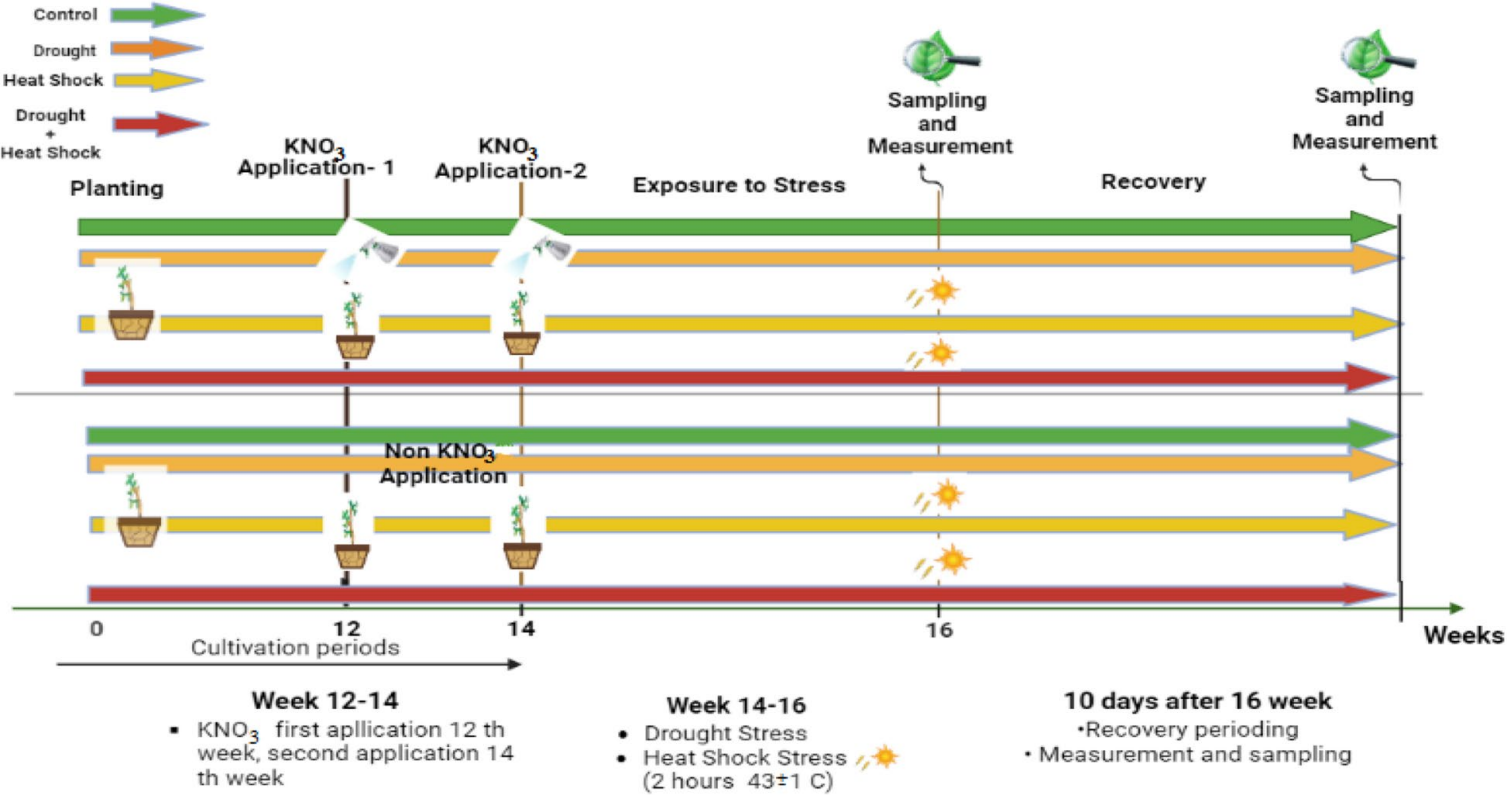
Experimental design to study the effect of KNO_3_ application, drought and heat shock stress in Myrobolan 29 C rootstock. Images created with BioRender.com

Leaf samples were gathered from both stress and recovery treatments, immediately flash-frozen in liquid nitrogen, and then stored at -80 °C for further analysis. After collecting the samples, the rootstocks were maintained under optimal growth conditions to evaluate their recovery capacity after stress. In addition to physiological parameters, the morphological parameters of the plants were examined. The pots were repositioned weekly within the greenhouse to avoid any potential effects on the plants. Figure 1 provides a visual representation of the experimental timeline and the different treatments applied to the Myrobalan 29C rootstocks.

### Relative shoot length,relative shoot diameter,plant fresh and dry weight measurements

After the recovery period, the plants underwent a comprehensive set of measurements and assessments to evaluate their overall size and dimensions. Relative shoot length (RSL) and relative shoot diameter (RSD) were meticulously measured using a ruler and digital calipers, providing valuable insights into the growth and development of the plants. The RSL was defined as the distance measured from the base of the shoot, just above the root crown, to the apical meristem at the shoot's tip. Similarly, the RSD was measured at a standardized point halfway up the shoot to ensure consistency across all samples. Following the methodology described by Bolat et al. [16], relative shoot diameter (RSD) and relative shoot length (RSL) measurements (%) were performed at the initiation of drought treatments and at the end of the trial. The increment (%) was calculated using the formula:

$$\mathrm{Increment}\;(\%)=(\mathrm{End}\;\mathrm{value}-\mathrm{Initial}\;\mathrm{value})/\mathrm{Initial}\;\mathrm{value})\;\times\;100$$

This approach allowed for a quantitative assessment of the changes in shoot diameter and length, highlighting the effects of the experimental conditions on plant growth and development. Subsequently, the plants were gently uprooted and rinsed with tap water to ensure the complete removal of any soil clinging to the roots. To determine the fresh root weight (FRW) and fresh shoot weight (FSW), an electronic balance was employed. This precise measurement technique allowed for the accurate assessment of the weight of both the roots and shoots, providing valuable information regarding the plant's biomass distribution and vigor. Subsequently, the plants were subjected to an oven-drying process at a temperature of 70 °C for a duration of 72 h. This drying process ensured the removal of all moisture from the plant tissues. Once completely dried, the plants underwent another weighing to assess the dry root weight (DRW) and the dry shoot weight (DSW).

### Determination of leaf area and leaf relative water content

To determine the leaf area (LA) per plant, both a leaf area meter and the ImageJ soft-ware program were employed. The leaf area meter facilitated the precise measurement of the total leaf surface area for each plant, offering valuable insights into their photosynthetic capacity and ability to assimilate nutrients. The average leaf area per plant was then calculated using the ImageJ software program, following the method outlined by Klam-kowski and Treder [17].This involved randomly marking leaves in the upper and middle segments of the shoot and quantifying the results in terms of the leaf area in square centimeters (cm^2^).

The leaf relative water content (LRWC) was assessed to examine the hydration status of the plant tissues. The LRWC is expressed as a percentage and was computed using the following equation, following the methodology of Yamasaki and Dillenburg [18].

$\mathrm{LRWC}\;(\%)=\lbrack(\mathrm{FW}-\mathrm{DW})/(\mathrm{TW}-\mathrm{DW})\rbrack\;\mathrm x\;100$, where FW represents fresh weight, DW represents dry weight, and TW represents turgid weight. The TW was obtained by subjecting the leaves to immersion in distilled water at a temperature of 4 °C for 24 h in the absence of light. By comparing the FW and DW of the leaf to the TW, the LRWC offers valuable insights into the moisture content and hydration status of the leaf tissue.

### Determination of stomatal conductance and chlorophyll content in leaves

To determine the stomatal conductance (SC) in leaves, measurements were taken between 12:00 and 14:00 h using a leaf Porometer (Model SC-1, Steady-State Diffusion Porometer, WA, USA). The measurement locations were marked on the upper and middle portions of the shoot, following the method described by Bolat et al. [16]. The leaf Porometer allowed for the assessment of SC, which provides insights into the leaf's ability to regulate gas exchange, particularly water vapor flux through stomatal openings.

Furthermore, the leaf chlorophyll content was quantified using a SPAD-502 Plus device (Konica Minolta Optics, Inc., Tokyo, Japan).The measurement locations were marked on the upper and apical parts of the shoot. The SPAD-502 Plus is a handheld device that measures the relative chlorophyll content in leaves based on the light absorption characteristics. The results obtained from the SPAD measurements were expressed as SPAD units, providing an indication of the chlorophyll concentration in the leaves. These measurements of stomatal conductance and chlorophyll content are crucial for assessing the physiological status of the plants, including their ability to perform photosynthesis and their overall health.

### Determination of proline, malondialdehyde (MDA), H_2_O_2_ content, and antioxidant enzymes in leaves

The proline content in the samples obtained from the treatments was calculated based on the methodology outlined by Bates et al. [19]. To prepare the sample, 0.5 g of leaf material was ground into a powder and combined with 10 mL of a 3% sulfosalicylic acid solution for this analysis. The resulting mixture went through a centrifuge for 10 min at 10,000 g. Once centrifugation was complete, 2 mL of the supernatant was added to tubes containing 2 mL of newly prepared acid-ninhydrin and 2 mL of glacial acetic acid. The tubes were subjected to a 1h incubation in a 90 °C water bath, after which they were extracted with 5 mL of toluene. After carefully collecting the toluene phase, the absorbance of the samples was read at 520 nm using a spectrophotometer (Shimadzu UV-1700, Kyoto, Japan). To calculate the proline content, L-proline was used to create a standard curve, and the absorbance results were reported as µg g^-1^ fresh weight.

Similarly, the changes in malondialdehyde (MDA) were measured with slight modifications tothe procedure out lined by Velikova et al. [20]. In this process, 0.5 g of the frozen sample was homogenized with TCA. Afterward, the mixture was subjected to centrifugation at 10,000 g for 5 min. The extract was carefully pipetted into tubes containing the reaction mixture of TBA and TCA. The tubes were then subjected to heating at 95 °C for 45 min, and subsequently cooled to halt the reaction. Following this, the tubes underwent centrifugation at 10,000 g for 15 min, and the absorbance values of the resulting supernatant were measured at 532 nm and 600 nm. The obtained results were expressed as nmol g^-1^ fresh weight.

To quantify the H_2_O_2_ content in the treatment samples, the protocol outlined by Kaya and Kose [21] was employed. The measurement of H_2_O_2_ was conducted using the eFOX reagent, while cold acetone containing 25 mM H_2_SO4 was utilized for sample extraction. Following centrifugation, the supernatant was combined with the eFOX reagent. After incubation and subsequent absorbance measurements at 550 nm and 800 nm, the H2O2 concentrations were calculated based on the standard slope graph prepared using known H2O2 amounts.

Additionally, for the analysis of antioxidant enzymes in the leaves, specifically superoxide dismutase (SOD), peroxidase (POD), and catalase (CAT), slight adjustments were measured to the protocols originally described by Beauchamp and Fridovich [22], Cvikrova et al. [23], and Aebi [24], respectively. The homogenization of 0.5 g samples was performed using 3 mL of 50 mM phosphate buffer (pH = 7) solution. After centrifugation at 15,000 × g for 15 min at 4 °C, the supernatant was stored at − 80 °C. The frozen sam-ples were powdered and extracted using 0.1 mM phosphate buffer (pH 7.8) containing 0.5% polyvinylpyrrolidone (PVP), 1 mMphenylmethylsulfonyl fluoride (PMSF), and 1 mM ethylenediaminetetraacetic acid (EDTA). The SOD, POD, and CAT activities were measured using spectrophotometry, with absorbance readings at 560 nm, 470 nm, and 240 nm, respectively. The enzyme activities were quantified in units (U), with 1 U of SOD activity defined as the enzyme amount required to reduce the absorbance measurement by 50%when compared to test bottles without the enzyme. Similarly, the activity of CAT and POD was quantified as 1 U, corresponding to the enzyme amount that resulted in a 0.01 increase in absorbance per minute.

### Hormone analysis of leaves

Extraction and purification of cytokinin, indole-3-acetic acid (IAA), gibberellic acid (GA), and abscisic acid (ABA) in the application samples were conducted with slight modifications to the methodology described by Kaya et al. [25]. This process aimed to analyze the levels of these plant hormones in the samples, providing insights into their involvement in various physiological processes.

To initiate the hormone analysis, 80% methanol was added to 1 g of fresh sample at -40 °C. The sample was then homogenized for 10 min to extract the hormones, and the obtained supernatants were subsequently filtered. To concentrate the hormone mixture in the samples, the filtrates were dried at 35 °C. The dried samples were then dissolved in a 0.1 M KH_2_PO_4_ (Potassium Dihydrogen Phosphate) solution with a pH of 8.0.For the separation and quantification of the hormones, a Sep-Pak C-18 cartridge (Waters) was employed. This cartridge facilitated the separation of the hormones from other compounds present in the samples. Subsequently, the hormones in the samples were determined using high-performance liquid chromatography (HPLC) with a UV detector (Agilent Technologies, Santa Clara, CA, USA). Measurement of the absorbance at 265 nm was employed for hormone detection.

### Mineral analysis of leaves

To analyze the nutrient contents of the harvested leaves, a standardized procedure was followed. First, the leaves were washed and dried to eliminate any impurities and excess moisture. The dried leaves were then subjected to a drying process in an oven to achieve a consistent weight suitable for analysis. After complete drying, grinding the leaves into a fine powder was achieved by using a mortar and pestle. This powdered form allowed for precise nutrient analysis. The leaf nitrogen content was assessed using the Kjeldahl procedure. This involved digesting the sample with concentrated sulfuric acid (H_2_SO_4_), followed by distillation and subsequent titration to precisely measure the nitrogen content.

To analyze the levels of magnesium, calcium, and potassium, an atomic absorption spectrophotometer was employed. To prepare the samples for analysis, the dried and ground leaf samples were subjected to a process called acid digestion. Acid digestion involves the breakdown of organic matter, which results in the release of the target elements into a solution for subsequent analysis. In this study, a mixture of nitric acid (HNO3) and hydrochloric acid (HCl) was used as the digestion solution. These concentrated acids are known for their ability to break down the complex organic matrix of the sample and facilitate the release of the target elements into solution. The acid digestion process began by adding a known volume of the acid mixture to the powdered leaf samples. The acid-sample mixture was then heated, typically using a hot plate or a digestion block, to promote the chemical reactions and breakdown of the organic matter. This heating step ensured the complete decomposition of the sample matrix and the solubilization of the Mg, Ca, and K elements. After digestion, the resulting solution containing the released elements was allowed to cool. It was then diluted with a suitable volume of deionized water to obtain a sample solution of appropriate concentration for analysis. The prepared sample solutions were then introduced into the atomic absorption spectrophotometer. Inside the instrument, a hollow cathode lamp emitting light at specific wavelengths for each element of interest (Mg, Ca, K) was employed.

The determination of phosphorus content was conducted using spectrophotometry, specifically with a Shimadzu UV-1700 spectrophotometer. This method relies on the formation of a colored complex between phosphorus and a reagent, which absorbs light at a specific wavelength. The absorbance of the resulting complex was measured, and the phosphorus content in the samples was calculated based on a standard curve. The methods used in this study’s analytical processes were those recommended by Kacar and Inal [26] for nutrient analysis in plant samples.

### Data analysis

The data obtained from the measurements of plant growth, and physiological and biochemical parameters were analyzed using SPSS software (SPSS Version 23, IBM, VA, USA). The mean values of each variable for each treatment group (control, drought, heat shock, drought + heat shock, and KNO3) were compared using a Tukey’s HSD analysis of variance (ANOVA). In the case of significant differences among the treatment groups, as indicated by ANOVA, the Tukey’s HSD test (*p* ≤ 0.01) was used to separate the means. The relationships among the variables were examined through Principal Component Analysis (PCA) based on the treatment groups, using the mean values of each variable for each group as the input data. Pearson correlation matrices of the variables were generated using R software (The R Foundation for Statistical Computing, Vienna, Austria).

## Results

### The effect of KNO_3_ application on some morphological parameters in leaves under stress and recovery

In our study, we investigated the effect of KNO_3_ application on certain morphological parameters in leaves during the recovery stage. Specifically, we analyzed the changes in relative shoot length (RSL), relative shoot diameter (RSD), fresh and dry shoot and root weights (FSW, DSW, FRW, DRW) and leaf area (LA) in both Non-KNO_3_ and KNO_3_-treated plants at harvest, considering the influence of different stress factors. Table 2 provides significant findings regarding the effects of drought, heat shock, and the combined stress of drought and heat shock (drought + heat shock) on these growth characteristics. The values of RSL, RSD, FSW, DSW, FRW, DRW, and LA exhibited notable decreases when exposed to the individual stresses of drought and heat shock, in contrast to the control treatment in both Non-KNO_3_ and KNO_3_-treated plants. This indicates that the combined stress had a negative effect on the growth parameters of the plants.

**Table 2:** Some morphological properties detected at harvest in leaves of Myrobalan 29C subjected to KNO_3_ and stress factors

**Stress factors**	**Applications**	**RSL (%)**	**RSD (%)**	**FSW** **(g/plant)**	**DSW** **(g/plant)**	**FRW** **(g/plant)**	**DRW** **(g/plant)**	**LA** **(cm**^**2**^**)**
Control	- KNO_3_	73.66^b^	33.66^b^	126.33_b_	81.66_b_	44.00^b^	29.33^b^	186.00^b^
Control	+ KNO_3_	79.33^a^	35.00^a^	151.00_a_	98.00_a_	48.00^a^	31.00^a^	233.00^a^
Drought	-KNO_3_	47.33^g^	18.66^c^	74.66_f_	49.00_e_	28.33^e^	18.00^e^	118.00^ef^
Drought	+ KNO_3_	54.33^e^	18.00^c^	80.66_e_	52.00_e_	31.00^d^	21.00^d^	134.00^d^
Heat Shock	-KNO_3_	62.33^d^	31.66^b^	85.33_d_	55.00_d_	39.00^c^	25.00^c^	127.00^de^
Heat Shock	+ KNO_3_	66.66^c^	31.33^b^	95.66_c_	61.00_c_	39.33^c^	25.00^c^	151.00^c^
Drought + Heat Shock	-KNO_3_	44.33^h^	16.33^c^	66.00_g_	42.00_g_	27.00^e^	13.00^f^	109.00^f^
Drought + Heat Shock	+ KNO_3_	50.33^f^	13.33^d^	76.33_f_	48.00_e_	30.33^d^	19.00^e^	127.00d^e^
Significance level		**	**	**	**	**	**	**

Different letters in columns represent statistical differences

*RSL* Relativeshootlength, *RSD* relativeshootdiameter, *FSW* freshshootweight, *DSW* dryshootweight, *FRW* freshrootweight, *DRW* dryrootweight and LAleaf area

^**^Significance level at *p* < 0.01 was detected for the applications and stress factors (Tukey’s HSD test)

However, we observed that the application of KNO_3_ alleviated the deleterious effects of the drought + heat shock stress on the morphological parameters. In KNO_3_-treated plants, under the influence of drought + heat shock stress, we observed an increase in RSL, FSW, DSW, FRW, DRW, and LA values compared to the Non-KNO3 plants. Specifically, the RSL, FSW, DSW, FRW, DRW, and LA values in KNO_3_-treated plants increased by 6%, 10.33 g, 6 g, 3.33 g, 6 g, and 18 cm^2^, respectively, compared to the Non-KNO3 plants. The only parameter that did not show a significant difference between the two groups was RSD. Similarly, under drought stress alone, the KNO3-treated plants exhibited higher values of RSL, FSW, DSW, FRW, DRW, and LA compared to the Non-KNO_3_ plants. The values of RSL, FSW, DSW, FRW, DRW, and LA in KNO_3_-treated plants increased by (7%, 6 g, 3 g, 2.66 g, 3 g, and 16 cm^2^, respectively, compared to the Non-KNO_3_ plants. Again, RSD did not show a significant difference between the two groups.

Regarding heat shock stress alone, the KNO_3_-treated rootstocks displayed higher values of RSL, FSW, DSW, and LA compared to the Non-KNO_3_ plants. The values of RSL, FSW, DSW, and LA in KNO_3_-treated plants increased by 4.33%, 10.33 g, 6 g, and 24 cm^2^, respectively, compared to the Non-KNO3 plants (Table 2). Overall, our findings indicate that the application of KNO_3_ positively influenced the morphological parameters of the plants during the recovery stage. It resulted in increased root length, shoot weight, root weight, and leaf area, particularly under drought, heat shock, and a combination of both. These results suggest that KNO_3_ application can enhance the recovery and growth potential of plants subjected to stressful environments, promoting their overall morphological development.

### The effect of KNO3 on plant physiological responses under stress and recovery

**Fig. 2:**
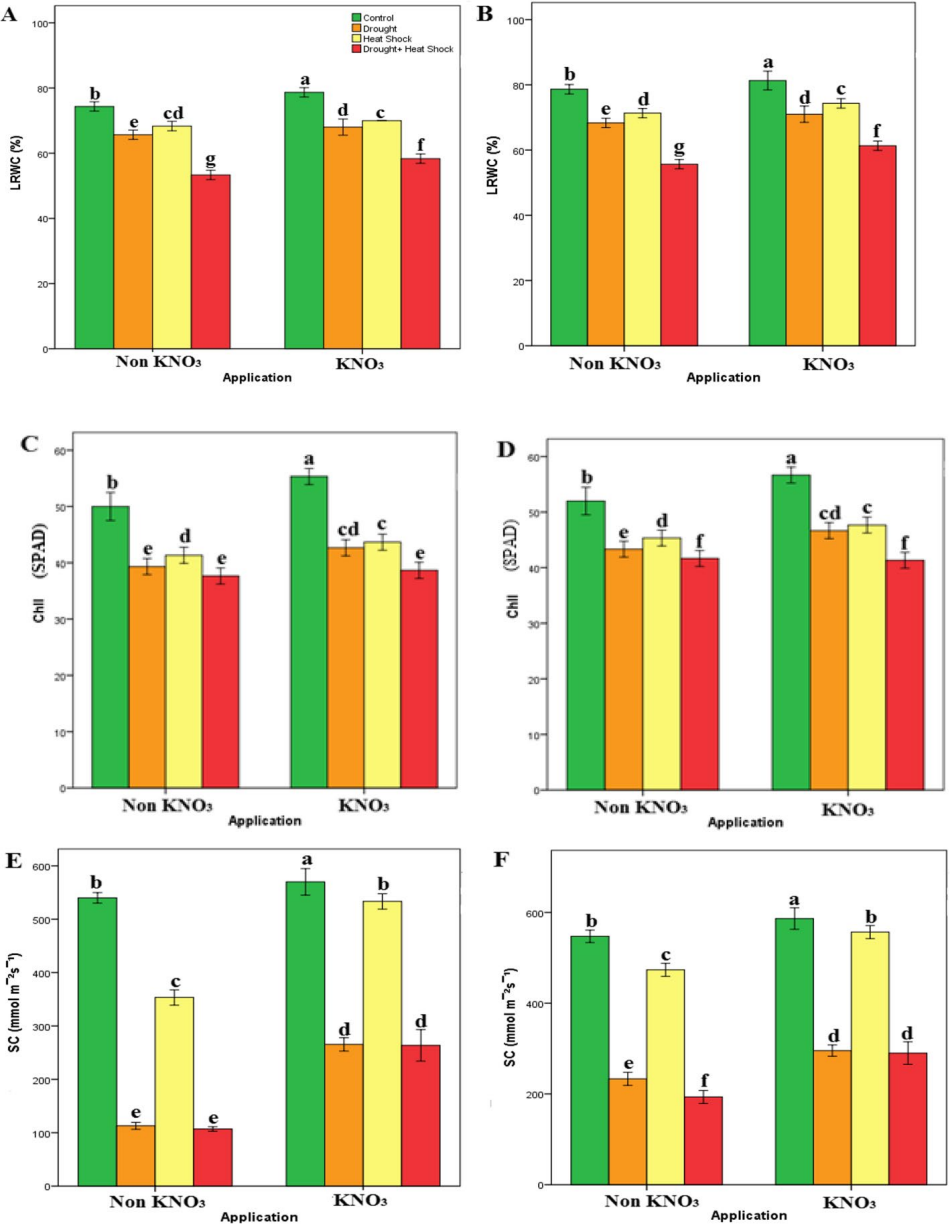
Some physiological parameters (LRWC, Chl, SC) in Myrobalan 29C during stress (**A**, **C** and **E**) and recovery periods (**B**, **D** and **F**). Lowercase letters indicate significance differences (Tukey’s HSD test; *p* ≤ 0.01) between applications and stress factors. Error bars on figures represent standard error (SE)

The study examined the effects of applying potassium nitrate (KNO_3_), a commonly used fertilizer, on crucial plant characteristics during periods of drought stress (DS), heat shock (HS), a combination of DS and HS (DS + HS), and the subsequent recovery phase. Figures 2A, C, and E presented the changes in Leaf Relative Water Content (LRWC), Chlorophyll (Chl), and Stomatal Conductance (SC) in Myrobalan 29C leaves during these stressful situations, while Figs. 2B, D, and F shed light on the recovery period that fol-lowed.

Significant variations were observed in the LRWC, Chl, and SC parameters among the rootstocks treated with different approaches during both the stress and recovery phases. In the control group, regardless of the KNO_3_ concentration (0% or 1%), the LRWC and Chl values were the highest, indicating superior water content and chlorophyll levels compared to the other treatment groups. Conversely, the rootstocks subjected to DS + HS treatments exhibited lower LRWC and Chl values, both during the stress period and the subsequent recovery phase.

During the stress treatment, both DS and HS alone led to significant reductions in LRWC, Chl, and SC values compared to the control group, highlighting severe stress effects on plant physiology. However, the application of KNO3 along with DS or HS partial-ly mitigated these effects, as evidenced by the higher values of LRWC, Chl, and SC observed compared to DS or HS alone.

The plants that underwent DS and HS together displayed the lowest values of SF, LRWC, Chl, and SC among all the treatment groups, indicating severe and synergistic stress effects on plant physiology. Nevertheless, the application of KNO_3_ along with DS + HS partially alleviated these effects, as indicated by the higher values of these parameters compared to DS + HS alone.

During the recovery stage, the control group maintained the highest values of LRWC, Chl, and SC among all the treatment groups, indicating successful plant recovery under normal conditions. Additionally, the application of KNO_3_ alone slightly increased these parameters compared to the control group, suggesting a sustained positive effect of KNO_3_ on plant physiology.

In contrast, plants exposed solely to drought stress and heat stress demonstrated notably reduced LRWC, Chl, and SC levels relative to the control group throughout the recovery phase, signifying suboptimal recovery under DS and HS conditions. Nonetheless, the introduction of KNO_3_ in conjunction with DS and HS individually showed a partial enhancement in these metrics during the recovery period when compared to the effects observed with DS and HS in isolation.

Similarly, the plants subjected to both DS and HS together had the lowest values of SF, LRWC, Chl, and SC among all the treatment groups during the recovery stage, indicating poor plant recovery under combined stress conditions. Nonetheless, the application of KNO_3_ along with DS and HS partially improved these parameters compared to DS and HS alone during the recovery stage.

### The effect of KNO_3_ application on proline, MDA, H_2_O_2_, and some antioxidant enzymes in leaves during drought stress, heat shock, combination of drought and heat shock and recovery

To elucidate the physiological effects of KNO_3_ application under stress and subsequent recovery, an in-depth analysis of stress-related parameters was conducted. Proline, MDA (malondialdehyde), H_2_O_2_ (hydrogen peroxide), CAT (catalase), SOD (superoxide dismutase), and POD (peroxidase) were examined as indicators of plant stress responses. The study aimed to assess the impact of different stress conditions, including drought, heat shock, and drought + heat shock, as well as the subsequent recovery phase. The primary focus was to understand the influence of two distinct KNO_3_ levels (0% and 1%) on these physiological parameters.

The experimental findings highlighted that, during the recovery phase, there were substantial changes in the measured parameters such as LRW), Chl, and SC in plants subjected to DS, HS, and the combined effect of DS + HS, in comparison to the control group. Notably, while plants exposed to DS and HS alone showed significantly lower values of LRWC, Chl, and SC, indicating a compromised recovery, the application of KNO_3_ to those under DS and HS conditions individually was observed to partially ameliorate these parameters during the recovery stage.Remarkably, KNO_3_-treated rootstocks exhibited distinct responses compared to non-treated ones under drought stress and recovery conditions. Among the evaluated treatments during stress and recovery, the combination of drought and heat shock stress in KNO_3_-treated rootstocks resulted in the highest levels of leaf proline, MDA, H_2_O_2_, CAT, SOD, and POD. Specifically, the measured values reached significant magnitudes of 0.40 mmol kg^−1^, 24 mmol kg^−1^, 38.3 mmol kg^−1^, 296 EU g^−1^ FW, 876 EU g^−1^ FW, and 12,628 EUg^−1^ FW, respectively, surpassing those observed in other treatments and the control group.

**Fig. 3:**
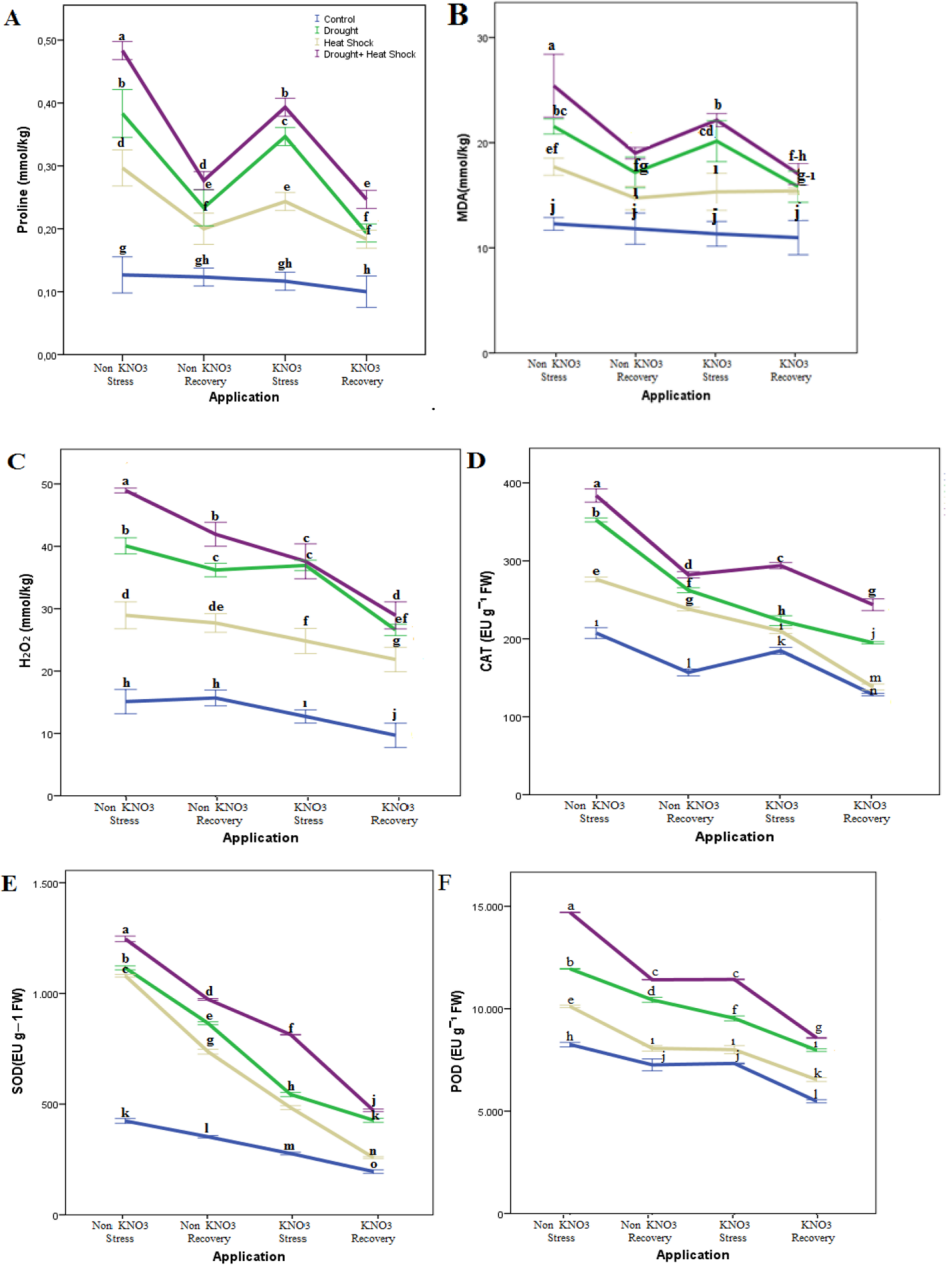
Some chemical parameters (Proline, MDA H_2_O_2_, CAT, POD, SOD) in Myrobalan 29C under applications and stress factors. Lowercase letters indicate significance differences (Tukey’s HSD test; *p* ≤ 0.01) between applications and stress factors. Error bars on figures represent standard error (SE)

Interestingly, the treatment history, which encompassed the previous exposure to various stress conditions, along with the application of KNO_3_, exerted significant influences on the final values of the physiological parameters measured at the end of the recovery period. During this crucial phase, the levels of leaf proline, MDA, H_2_O_2_, CAT, SOD, and POD were observed to be at their lowest compared to the levels observed in plants subjected to non-KNO_3_ stress or recovery conditions (as depicted in Fig. 3). These findings underscore the role of KNO_3_ in mitigating physiological stress responses during the recovery period, thus highlighting its potential in promoting plant recovery and resilience.

### The effect of KNO_3_ application on hormone content in leaves during drought stress, heat shock, combination of drought and heat shock and recovery

Significant differences were observed in the hormonal responses of plants treated with different combinations of KNO_3_, DS, and HS alone or the combination of drought and heat shock stress. The results are summarized in Table 3. During both the stress and recovery stages, the control group exhibited the highest values of IAA, GA and cytokinin, while displaying the lowest value of ABA. These results indicate higher levels of growth hormones and a lower level of stress hormone under normal conditions. Notably, the application of potassium nitrate (KNO_3_) alone slightly increased the levels of IAA, GA, and cytokinin by 90, 130, and 194%, respectively, while decreasing the level of ABA by 47%, compared to the control group. This suggests a positive influence of KNO_3_ on plant hormonal responses. In contrast, the plants exposed to drought stress (DS) and heat shock (HS) alone exhibited significantly lower values of IAA, GA, and cytokinin, along with higher levels of ABA, compared to the control group. These findings indicate reduced levels of growth hormones and an elevated level of stress hormone under drought conditions. However, when KNO_3_ was applied along with DS and HS alone, there was a partial alleviation of these effects. This was evident from the higher values of IAA, GA, and cytokinin, which increased by 108, 127, and 119%, respectively, compared to DS alone. Additionally, the application of KNO_3_ resulted in a lower value of ABA, which decreased by 57% compared to DS alone. Similarly, compared to HS alone, the levels of IAA, GA, and cytokinin increased by 151, 157, and 21%, respectively, while the value of ABA decreased by 56%. These results suggest that the addition of KNO_3_ can help mitigate the negative impact of DS and HS on plant hormonal responses.

**Table 3:** Effect of KNO_3_ and stress factors on hormone contents in Myrobolan rootstock

Stress factors	Applications	IAA (ng.mg^−1^tissue)	ABA ng.g^−1^D.W	GA ng.g^−1^D.W	Cytokinin ng.g^−1^D.W
		Stress			
Control	-KNO_3_	1.22^b^	281.00^e^	1.16^c^	1.90^bc^
Control	+ KNO_3_	2.32^a^	152.00^f^	2.67^a^	5.59^a^
Drought	-KNO_3_	0.25^f^	1099.66^b^	0.54^d^	0.62^f^
Drought	‘KNO_3_	0.52^d^	481.00^d^	1.23^c^	1.36^c^
Heat Shock	-KNO_3_	0.27^f^	650.00^c^	0.63^d^	0.76^e^
Heat Shock	+ KNO_3_	0.68^c^	285.66^e^	1.62^b^	0.92^d^
Drought + Heat Shock	-KNO_3_	0.16^g^	1446.66^a^	0.31^e^	0.45^g^
Drought + Heat Shock	+ KNO_3_	0.43^e^	569.00c	1.14^c^	2.45^b^
Significance level		**	**	**	**
		Recovery			
Control	-KNO_3_	1.41^c^	257.33^e^	1.41^e^	2.11^d^
Control	+ KNO_3_	2.69^a^	135.66^f^	5.52^a^	6.95^a^
Drought	-KNO_3_	0.34^g^	767.00^b^	0.88^f^	0.79^g^
Drought	‘KNO_3_	1.455^c^	269.66^e^	2.35^d^	3.68^c^
Heat Shock	-KNO_3_	0.54^e^	557.00^c^	1.09^ef^	0.95^f^
Heat Shock	+ KNO_3_	1.61^b^	204.66^ef^	4.29^b^	4.21^b^
Drought + Heat Shock	-KNO_3_	0.42^f^	855.66^a^	0.48^g^	0.58^h^
Drought + Heat Shock	+ KNO_3_	1.02^d^	352.66^d^	3.47^c^	1.89^e^
Significance level		**	**	**	**

Different letters in columns represent statistical differences

^**^Significance level at *p* ≤ 0.01 was detected for the applications and stress factors (Tukey’s HSD test)

Furthermore, the plants subjected to both DS and HS together demonstrated the lowest values of IAA, GA, and cytokinin, accompanied by the highest level of ABA among all the treatment groups. This indicates reduced levels of growth hormones and the highest level of stress hormone under combined stress conditions. However, when KNO_3_ was applied along with DS and HS together, there was a partial alleviation of these effects. This was evidenced by the higher values of growth hormones by 168, 267, and 444% for IAA, GA, and cytokinin, respectively, and the lower level of stress hormone by 61%, compared to DS and HS together. These results highlight the potential of KNO_3_ in modulating plant hormonal responses and attenuating the adverse effects of combined DS and HS stress on plant hormonal balance.

### The effect of KNO_3_ application on the mineral content in leaves during drought stress, heat shock, combination of drought and heat shock and recovery

The analysis revealed that both DS and HS, as well as DS + HS, significantly reduced the efficiency of nutrient uptake in the plants. Specifically, the levels of N, Ca, K, Mg, P, Mn, Fe, and Zn were consistently and significantly reduced under the influence of these stress factors. However, the severity of this effect varied among the different stress conditions (Table 4). A comparison between the Non-KNO_3_ and KNO_3_-treated plants showed that the lowest levels of K, P, N, Ca, Fe, Mn, Mg, and Zn were observed in samples exposed to DS + HS stress, both during the stress and recovery periods. This was followed by samples exposed to DS and HS individually. These findings indicate that the combined stress of DS + HS had the most detrimental impact on the mineral content of the leaves.

**Table 4:** Effect ofKNO_3_ and stress factors on mineral contents in Myrobolan rootstock

Stress factors	Applications	N (%)	P (%)	K (%)	Ca (%)	Mg (%)	Fe (ppm)	Mn (ppm)	Zn (ppm)
				Stress					
Control	-KNO_3_	2.33^b^	0.25^a^	1.55^a^	1.21^a^	0.22^c^	27.44^e^	21.71^d^	17.95^b^
Control	+ KNO_3_	2.85^a^	0.22^b^	1.78^a^	1.25^a^	0.49^a^	71.04^a^	57.56^a^	21.01^a^
Drought	-KNO_3_	1.34^d^	0.17^cd^	1.12^b^	0.72^b^	0.13^e^	17.15^f^	16.33^ef^	11.67^d^
Drought	‘KNO_3_	2.34^b^	0.17^cd^	1.26^b^	0.68^b^	0.34^b^	45.21^c^	44.79c	14.71^c^
Heat Shock	-KNO_3_	1.59^cd^	0.17^cd^	1.26^b^	0.77^b^	0.17^d^	24.33^e^	17.19^e^	14.23_c_
Heat Shock	+ KNO_3_	2.53^ab^	0.18^c^	1.61^a^	0.89^b^	0.45^a^	54.32^b^	51.60^b^	17.78^b^
Drought + Heat Shock	-KNO_3_	0.92^e^	0.13^e^	1.06^b^	0.66^b^	0.12^e^	15.59^f^	14.38^f^	9.20^e^
Drought + Heat Shock	+ KNO_3_	1.88^c^	0.15^de^	1.14^b^	0.80^b^	0.26^c^	33.20^d^	42.84^c^	13.76^cd^
Significance level		**	**	**	**	**	**	**	**
Recovery									
Control	-KNO_3_	2.28^b^	0.13^a^	1.11a	0.98^ab^	0.23^cd^	31.86^d^	25.12^e^	24.19^b^
Control	+ KNO_3_	2.64^a^	0.21^a^	0.92b	0.99^a^	0.54^a^	74.49^a^	62.24^a^	29.65^a^
Drought	-KNO_3_	1.52^e^	0.16^b^	0.82^cd^	0.89^c^	0.13^ef^	22.12^e^	18.37^fg^	16.63^d^
Drought	‘KNO_3_	2.17^bc^	0.13^bc^	0.76d	0.64^e^	0.37^b^	52.11^b^	48.21^c^	19.50^c^
Heat Shock	-KNO_3_	1.94^c^	0.14^bc^	0.94b	0.90^b^	0.18^de^	29.52^d^	20.42^f^	19.79^c^
Heat Shock	+ KNO_3_	2.44^ab^	0.14^bc^	0.86bc	0.87^c^	0.41^b^	57.73^b^	56.92^b^	24.46^b^
Drought + Heat Shock	-KNO_3_	1.30^f^	0.12^bc^	0.73de	0.72^d^	0.12^f^	26.76^de^	16.10^g^	14.45^e^
Drought + Heat Shock	+ KNO_3_	1.75^d^	0.11^c^	0.65e	0.71^d^	0.28^c^	43.21^c^	45.44^d^	17.49^d^
Significance level		**	**	**	**	**	**	**	**

Different letters in columns represent statistical differences

^**^Significance level at *p* ≤ 0.01 was detected for the applications and stress factors (Tukey’s HSD test)

Furthermore, clear upward trends in the levels of N, Ca, K, P, Fe, Mg, Zn, and Mn were observed in the KNO_3_-treated plants compared to the Non-KNO_3_ plants during both the stress and recovery periods This suggests that KNO_3_ application had a positive influence on the nutrient status of the plants, particularly in terms of macro- and micronutrients.

Overall, the results demonstrate that stress conditions, including DS, HS, and DS + HS, lead to significant reductions in the mineral content of leaves. However, the application of KNO_3_ alleviated these negative effects by promoting an increase in nutrient levels during both stress and recovery periods. This highlights the potential of KNO_3_ as a beneficial treatment for enhancing nutrient uptake and maintaining optimal mineral levels in plants subjected to stressful conditions.

### General evaluation of physiological responses

**Fig. 4:**
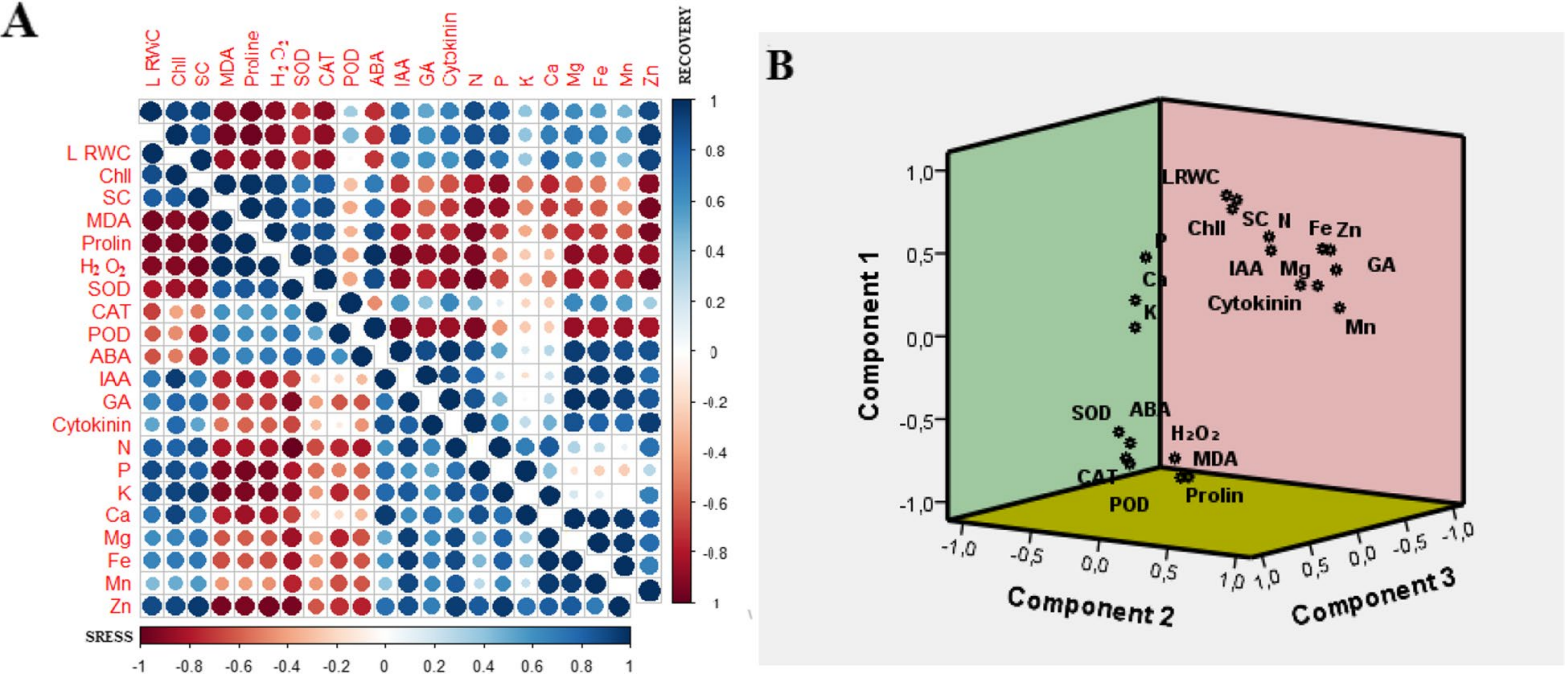
**A** Correlation analysis **B** Three-dimensional PCA plot of the 21 quantitative traits with regard to according to the first three main components on Myrobalan 29C

Considering complete data sets for general evaluation of physiological responses, one showing the relative physiological parameters under recovery and another showing these changes indicating under drought stress, heat shock, combination of drought and heat shock were subjected to the Pearson correlation coefficient and Principal component analysis (PCA) (Fig. 4 A and B). Depicting responses to drought stress, as shown in Fig. 4A, LRWC, Chll, SC, IAA, GA, Cytokinin, N, Ca, K, Mg, P, Mn Fe, Zn were positively correlated with each other. Similarly, MDA, proline, H_2_O_2_, SOD, CAT, POD, ABA were positively correlated, whereas these parameters were negative for LRWC, Chll, SC, IAA, GA, Cytokinin, N, Ca, K, Mg, P, Mn Fe, Zn was related. Depicting responses to recovery, on the other hand, MDA, H_2_O_2_, SOD, POD, ABA Chll, SC, were negatively correlated with IAA, GA, Cytokinin and minerals, whereas these parameters were positively correlated with ABA, CAT, GA. Besides, IAA, GA, Cytokinin, ABA, N, P, K, Ca, Zn were positively correlated with each other (Fig. 4A). Regarding PCA, Component 1 was heavily associated with Ca, P, K, ABA, CAT and SOD; Component 2 gave a high weighting to POD and prolin, and Component 2 was associated with LRWC, Chll, SC, IAA, N, Cytokinin, Mg, Fe, Zn, GA, Mn, H_2_O_2_ and MDA (Fig. 4B).

## Discussion

Heat and drought stress are major challenges for fruit plants that grow in arid or temperate climates [2, 27]. These plants have to cope with harsh conditions, but they also have the ability to recover when the situation improves. This research aims to understand how these plants survive and adapt to these stresses, and how they can benefit other crops that face similar problems [16, 28, 29]. One of the specific objectives of this study is to examine the effect of KNO_3_ application on the resistance and recovery of Myrobalan 29C rootstocks, which are widely used for fruit production. By doing so, this study hopes to reveal new insights into the mechanisms and potential of these rootstocks for a sustainable future.

### The effect of KNO_3_ application on some morphological parameters in leaves under the stress and recovery

Morphological parameters, namely, root length and weight, shoot weight, and leaf area, reflect the ability of plants to acquire and utilize resources such as water, nutrients and light, which are essential for plant growth and development [30, 31]. However, these parameters are reduced by drought and heat shock, which impair plant morphology by affecting cell division, expansion, differentiation and senescence [32, 33]. Therefore, improving plant morphology under stress conditions is a crucial strategy for enhancing plant performance and adaptation [34]. In this study, we observed that drought, heat shock and their combination reduced the morphological parameters of rootstocks compared to the control treatment. However, KNO_3_-treated rootstocks showed higher morphological parameters than non-treated ones under stress and recovery conditions. This indicates that KNO_3_ application improved plant morphology under stress conditions by providing additional sources of K and N that are essential for plant growth and development [35, 36]. Moreover, potassium and nitrogen application enhanced plant morphology by stimulating cell division, expansion, differentiation, and senescence [37–39]. Our findings align with previous studies that showed the positive effects of KNO_3_ application on plant morphology under various abiotic stresses such as drought [40], heat [41] and waterlogging stress [36]. However, there seems to be no study investigating the effects of KNO_3_ application on plant recovery after stress relief. Therefore, our study offers novel insights into the effects of KNO_3_ application in improving plant morphology under stress conditions and during recovery.

### The effect of KNO_3_ on some key plant physiological parameters, proline, MDA, H_2_O_2_, and some antioxidant enzymes under stress and recovery

Physiological parameters such as leaf relative water content (LRWC), chlorophyll content (Chl), stomatal conductance (SC), proline content (Pro), hydrogen peroxide content (H_2_O_2_), malondialdehyde content (MDA) and antioxidant enzyme activities (SOD, CAT, POD) indicate the water status, photosynthetic capacity and oxidative stress level of plants under abiotic stresses. The impact of drought and heat stress on these parameters varies depending on the intensity and duration of the stress. Generally, drought and heat stress reduce LRWC, Chl and SC by causing water loss, chlorophyll degradation and stomatal closure in plants [42–44]. This leads to reduced photosynthesis efficiency and increased production of ROS such as H_2_O_2_ [45]. ROS can harm cellular components, including lipids, proteins, and nucleic acids, leading to an elevation in MDA content, which serves as an indicator of lipid peroxidation [46]. To cope with oxidative stress, plants activate antioxidant enzymes such as SOD, CAT and POD to scavenge ROS [47]. Plants also accumulate osmolytes such as proline to maintain osmotic balance and protect cellular structures from dehydration [48]. In this study, we observed that drought, heat shock and their combination reduced LRWC, Chl, SC, Pro, SOD, CAT, POD parameters and increased H_2_O_2_, MDA parameters in non-treated rootstocks compared to the control treatment. However, rootstocks treated with KNO_3_ exhibited higher LRWC, Chl, SC, Pro, SOD, CAT, and POD parameters, along with lower H_2_O_2_ and MDA parameters, than non-treated rootstocks under stress and recovery conditions. The application of KNO_3_ plays a significant role in influencing the physiological bases of heat shock and drought resistance, as well as subsequent recovery, by reducing damage to parameters such as LRWC, Chl, SC, proline, H_2_O_2_, SOD, CAT, POD, and lipid peroxidation. Similar findings have been reported by Jan et al. [49] in sunflower, demonstrating increased contents of LRWC, Chl, SC, proline, SOD, CAT, POD, and decreased contents of H_2_O_2_ and MDA under drought stress with KNO_3_ addition. Other studies have also supported these findings, highlighting the positive effects of exogenous KNO_3_ application on photosynthetic performance, membrane lipid peroxidation reduction, and ROS accumulation alleviation during drought stress [50]. Additionally, nitrogen supplementation, such as KNO_3_, has been shown to mitigate membrane lipid damage caused by peroxide and maintain enzymatic activities, including SOD, CAT, and POD, under drought stress conditions [51]. Moreover, our results indicate that non-KNO_3_-treated rootstocks had increased MDA content under stress conditions and recovery periods compared to KNO_3_-treated rootstocks, implying ROS accumulation as the likely cause.

Furthermore, the application of KNO_3_ enhances the physiological status of rootstocks under stress conditions by providing additional sources of potassium (K) and nitrogen (N), which are essential for osmotic regulation, stomatal movement, enzyme activation, membrane stability, and chlorophyll formation [52, 53]. These findings align with previous research demonstrating the beneficial effects of KNO_3_ application in mitigating oxidative stress and enhancing the antioxidant defense system under numerous abiotic stresses, such as drought and salinity [50, 54, 55]. The positive impact of KNO_3_ application on drought tolerance can be attributed to its ability to facilitate osmotic adjustment, maintaining turgor pressure, and improving water use efficiency, osmoregulation, and cell membrane integrity [13, 56]. Moreover, the present study demonstrates that KNO_3_ application not only improves plant physiology during stress exposure but also enhances plant recovery after stress removal, indicating its potential in priming plants for future stresses and enhancing their cross-stress tolerance.

### The effect of KNO_3_ application on hormone content in leaves during drought stress, heat shock, combination of drought and heat shock and recovery

Hormones, among other biochemical parameters, are crucial for governing plant growth and development in both favorable and challenging conditions [57]. They participate in diverse processes, including signal transduction, gene expression, enzyme activity, cell division, cell differentiation, cell elongation, cell senescence [58, 59]. Under abiotic stresses like drought and heat, various parameters related to plant hormones undergo changes [60]. This includes alterations in biosynthesis, transport, metabolism, and crosstalk between these important factors. As a consequence, plant growth, development, and adaptation can be significantly affected [59]. To enhance plant performance and adaptation, it is essential to modulate the levels of hormones in response to stressful conditions [61, 62]. In this study, we observed that drought, heat shock, and their combination affected the levels of hormones such as IAA, ABA, GA, and cytokinin (CK) in rootstocks compared to the control treatment. Generally, drought and heat stress reduced IAA, GA, and CK levels by affecting their biosynthesis, transport, metabolism, and crosstalk in plants. This leads to reduced cell division, cell differentiation, cell elongation and cell senescence [63, 64]. On the other hand, drought and heat stress activate the biosynthesis, transport, metabolism, and crosstalk of abscisic acid (ABA) in plants, increasing its levels, which in turn regulates stomatal closure to mitigate water loss from the leaf surface [65, 66]. However, KNO_3_-treated rootstocks showed higher IAA, GA, and CK levels and lower ABA level than non-treated ones under stress and recovery conditions. This indicates that KNO_3_ application modulated the levels of hormones under stress conditions by providing additional sources of K and N that are involved in hormone biosynthesis, transport, metabolism, and crosstalk in plants [67]. Our results are consistent with previous studies that showed the positive effects of KNO_3_ application on plant hormones under various abiotic stresses [68].

### Investigating the impact of KNO_3_ application on leaf mineral content under drought, heat shock, combined stress, and recovery conditions

In addition to hormones, minerals also play vital roles in plant growth and stress responses. Minerals including N, P, K, Ca, Mg, Fe, Zn etc. are essential for various metabolic processes, enzyme activities, membrane stability, photosynthesis and redox balance etc. in plants [69]. Under abiotic stresses, the availability and uptake of these minerals can be affected, leading to nutrient deficiency and imbalance in plants [58]. Therefore, it is important to maintain optimal levels of minerals in plants under stress conditions to enhance their growth and adaptation [59]. In this study, we measured the levels of N, P, K, Ca, Mg, Fe and Zn in rootstocks under different treatments. We found that drought and heat stress reduced the levels of these minerals in rootstocks compared to the control treatment. This could be due to reduced soil moisture, root activity, transpiration rate and nutrient transport under stress conditions [70, 71]. However, KNO_3_-treated rootstocks showed higher levels of these minerals than non-treated ones under stress and recovery conditions. Our results are in agreement with previous studies that showed the beneficial effects of KNO_3_ application on plant mineral nutrition under various abiotic stresses such as drought [29, 72], salinity [54, 73] and heat [41].

The present study also demonstrated that KNO_3_ application not only improved plant hormones during stress exposure but also facilitated plant recovery after stress removal. The interaction between hormones and minerals is complex and dynamic in plants under abiotic stress conditions. Hormones can regulate the uptake, transport and distribution of minerals in plants by modulating root architecture, membrane permeability, ion channels and transporters [74]. Conversely, minerals can affect the biosynthesis, metabolism and signalling of hormones in plants by acting as cofactors or substrates for enzymes involved in hormone pathways [75]. Therefore, it is essential to understand the crosstalk between hormones and minerals in plants under stress conditions to elucidate the molecular mechanisms of plant adaptation and tolerance [60].

### Mechanistic model

**Fig. 5:**
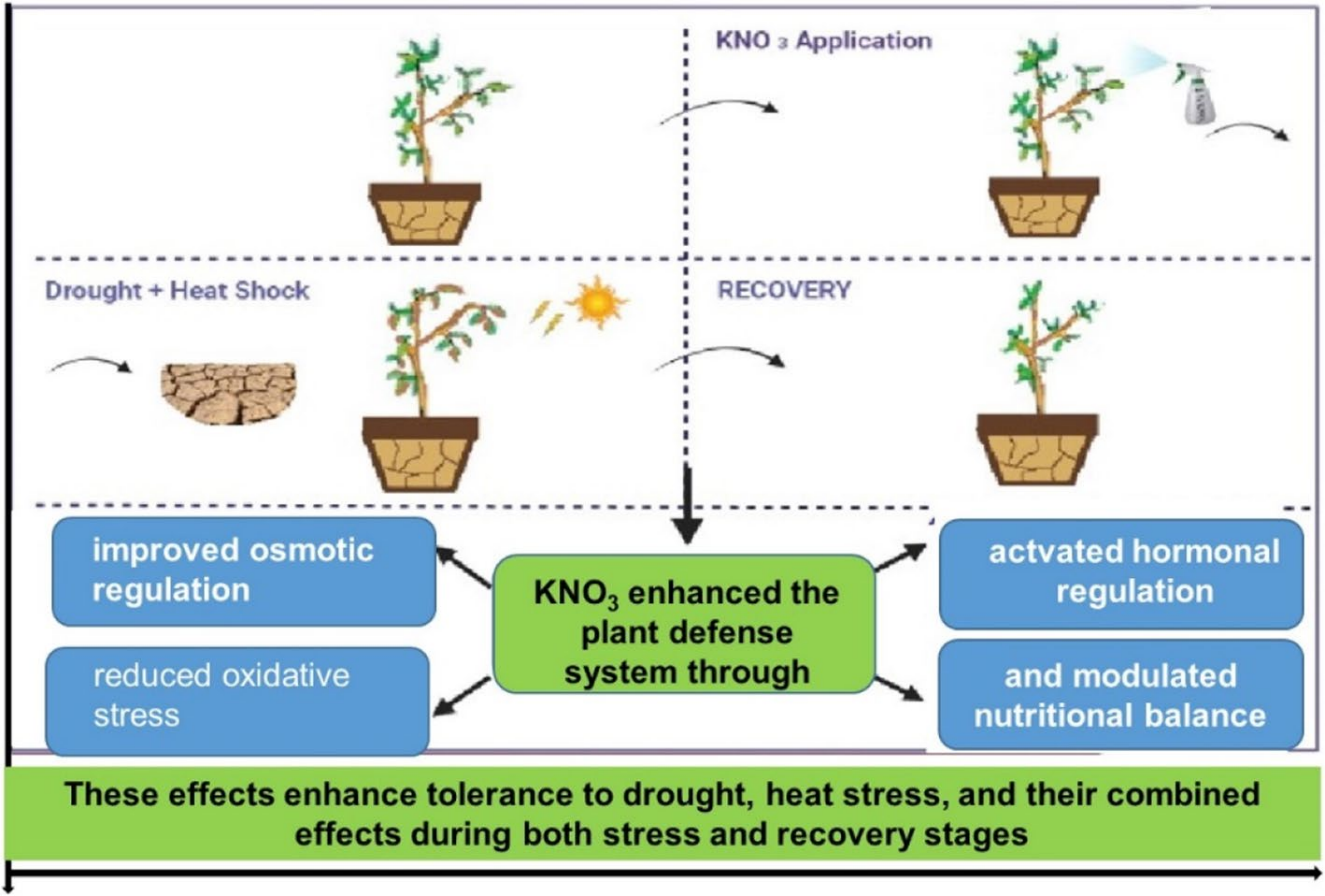
Mechanisms underlying KNO_3_-mediated drought, heat stress, and combined stress tolerance in Myrobalan 29C rootstocks during both stress and recovery stages

Mechanism of KNO_3_-mediated drought, heat, and combined stress tolerance in plants (Fig. 5):

- Osmotic regulation: KNO_3_ helps to improve osmotic regulation by increasing the osmotic pressure of the cell sap. This makes it more difficult for water to leave the cells and helps to protect them from damage.
- Antioxidant defense: KNO_3_ helps to reduce oxidative stress by increasing the levels of antioxidants in plants. Antioxidants are molecules that can neutralize free radicals and protect cells from damage.

Potassium and Nitrate (NO_3_^−^) play pivotal roles in plant stress responses, with potassium being integral to osmoregulation, stomatal movement, and enzyme activation, all critical for stress tolerance. Nitrate acts as a primary nitrogen source essential for amino acid and protein synthesis, including those proteins involved in hormone biosynthesis and stress response mechanisms. As such, KNO_3_ supplementation provides essential nutrients that bolster plant growth and resilience to stress [76]. Concurrently, growth hormones such as IAA, GA, and cytokinins, which facilitate cell division and growth, are upregulated. This upregulation aids in sustaining growth under stress conditions, counterbalancing the effects of ABA, a hormone that mediates stress responses by inducing stomatal closure to mitigate water loss but can also inhibit growth when overly abundant. Modulating the plant's hormonal milieu towards growth-promoting hormones while diminishing ABA levels can therefore enhance the plant's growth capacity under stress by striking a balance between stress defense and growth [77]. The controversy around reducing ABA levels to boost stress resistance stems from oversimplifying ABA's critical role in stress adaptation. Nonetheless, the application of KNO_3_ might ameliorate plant nutritional status and water use efficiency, reducing dependency on ABA-mediated responses. This nuanced approach suggests that a moderate decrease in ABA, coupled with increased levels of growth-promoting hormones, does not undermine ABA's significance in stress tolerance. Instead, it signifies a well-nourished plant capable of balancing growth with efficient stress management [78]. This perspective underscores the complexity of plant hormonal interactions and nutrient management in enhancing plant stress resilience.

- Nutritional balance: KNO_3_ helps to modulate nutritional balance by providing plants with potassium, which is an essential nutrient for plant growth. Potassium and nitrogen help plants produce chlorophyll, which is necessary for photosynthesis. They also help plants to transport water and nutrients throughout the plant and to defend themselves against stress.

Overall, KNO_3_ can help plants to tolerate drought, heat stress,and combined stress by improving osmotic regulation, reducing oxidative stress, activating hormonal regulation, and modulating nutritional balance.

## Conclusions

The impact of KNO_3_ application on the physiological and hormonal responses of rootstocks was investigated in the presence of drought, heat shock, and drought + heat shock stress, as well as during the recovery period. The results demonstrated that the application of KNO_3_ improved the stress tolerance and recovery of rootstocks. This improvement was achieved through the modulation of various parameters, including proline, MDA, H_2_O_2_, antioxidant enzymes, IAA, GA, cytokinin, ABA, and mineral nutrients in the leaves. The findings indicate that KNO_3_ application has the potential to enhance plant performance and resilience in the face of changing climatic conditions. This enhancement occurs through the regulation of multiple pathways and processes involved in plant growth and stress adaptation. This study contributes to the current knowledge on the role and mechanisms of KNO_3_ in plant stress responses and provides new insights into the potential of KNO_3_ as a beneficial treatment for enhancing food security. However, additional investigations are warranted to clarify the molecular mechanisms involved in KNO_3_-mediated signalling and gene expression in plants under abiotic stresses. Unravelling the molecular mechanisms of KNO_3_-induced stress memory will provide new insights into the regulation of plant stress responses and adaptation. This will also facilitate the development of novel tools and methods for improving crop resilience and adaptation to changing climatic conditions. Further research on this topic will advance the field of plant stress biology. Additionally, the knowledge on rootstock/scion interaction of this rootstock may be useful as parent in future breeding programs.

## Data Availability

Correspondence and requests for materials should be addressed to O.K.
